# Draft Genome Sequences for Three Mercury-Methylating, Sulfate-Reducing Bacteria

**DOI:** 10.1128/genomeA.00618-13

**Published:** 2013-08-15

**Authors:** Steven D. Brown, Richard A. Hurt, Cynthia C. Gilmour, Dwayne A. Elias

**Affiliations:** Biosciences Division, Oak Ridge National Laboratory, Oak Ridge, Tennessee, USAa; Smithsonian Environmental Research Center, Edgewater, Maryland, USAb

## Abstract

The genetic basis for bacterial mercury methylation has been described recently. For insights into the physiology of mercury-methylating bacteria, we present genome sequences for *Desulfococcus multivorans* strain DSM 2059, *Desulfovibrio alkalitolerans* strain DSM 16529, and *Desulfovibrio* species strain X2.

## GENOME ANNOUNCEMENT

Select members of the sulfate-reducing bacteria (SRB) and Fe(III)-reducing bacteria (IRB) can methylate inorganic mercury [Hg(II)] to methylmercury (MeHg), a more toxic form that bioaccumulates (1). For the first time, recent genetic studies have linked a specific gene cluster (*hgcAB*) to mercury methylation in the model SRB *Desulfovibrio desulfuricans* strain ND132 and IRB *Geobacter sulfureducens* strain PCA (2). The *hgcAB* gene cluster encodes a putative corrinoid-containing CO dehydrogenase/acetyl-coenzyme A (CoA) synthase, HgcA, and a 2[4Fe-4S] ferredoxin, HgcB, and these are predicted to have roles as a methyl carrier and an electron donor, respectively.

The potential involvement of the acetyl-CoA pathway in bacterial Hg methylation was recognized more than 20 years ago (3), although the physiology and MeHg formation in incomplete-oxidizing SRB (conversion of carbon substrates to acetate) and complete-oxidizing SRB (conversion of carbon to CO_2_) remain to be fully elucidated (4, 5). The majority of known Hg-methylating *Deltaproteobacteria* are incomplete oxidizers (6). This means that when they reduce sulfate, these organisms incompletely oxidize short-chain fatty acids to acetate and do not utilize C-1 substrates, and thus they do not use the acetyl-CoA pathway as part of their primary substrate utilization machinery during heterotrophic growth (7). Detailed assessments and comparisons of mercury methylation rates for several *Desulfovibrio* species that are incomplete oxidizers, including those investigated in this study, have been reported (6, 8). Genome sequences for several SRBs known or predicted to be mercury methylators have been reported (2, 9–12).

*Desulfovibrio multivorans* is an example of a complete oxidizer with an acetyl-CoA pathway, and mercury methylation rates have been reported for several strains (4, 5). *D. alkalitolerans* strain DSM 16529 is an example of a complete oxidizer that can generate MeHg (8). *Desulfovibrio* species strain X2 was isolated from soft black mid-Chesapeake Bay bottom sediments sampled in May 1985 (13), and it methylates both Sn(IV) (14) and Hg(II) (6), but its detailed physiology has not been fully assessed.

In this study, we generated draft genomes for *D. multivorans* DSM 2059, *D. alkalitolerans* DSM 16529, and *Desulfovibrio* species strain X2. Sequence data for each genome were generated using an Illumina MiSeq instrument (15) and a paired-end approach with an approximate insert library size of 500 bp and read lengths of 250 bp, according to the manufacturer’s instructions. The CLC Genomics Workbench (version 6.0.2) was used to trim and filter reads for quality sequence data and subsequent assemblies. The resulting assemblies generated 149, 32, and 66 DNA contigs that represented the draft genome sequences for strains DSM 2059, DSM 16529, and X2, respectively. The estimated genome sizes were ~4.4, 3.2, and 3.9 Mb and G+C DNA contents were 56.8%, 64.4%, and 67.9%, and there were 3,838, 2,924, and 3,445 putative coding sequences (CDS) for DSM 2059, DSM 16529, and X2, respectively. Sequence depth coverage across the genomes was ~205 to 309 times the genome sizes. Draft genome sequences were annotated as previously described (16).

The draft genome sequences presented here will facilitate comparative studies and assist with investigations into HgcAB native functions, related pathways, and assessments of MeHg potential in different ecological niches.

### Nucleotide sequence accession numbers.

This whole-genome shotgun project has been deposited at DDBJ/EMBL/GenBank under the accession numbers ATHJ00000000, ATHI00000000, and ATHV00000000 for *D. multivorans* DSM 2059, *D. alkalitolerans* DSM 16529, and *Desulfovibrio* species strain X2, respectively. The versions described in this paper are the first versions.
